# Positive Virological Outcomes of HIV-Infected Patients on Protease Inhibitor-Based Second-Line Regimen in Cambodia: The ANRS 12276 2PICAM Study

**DOI:** 10.3389/fpubh.2018.00063

**Published:** 2018-03-19

**Authors:** Olivier Ségéral, Eric Nerrienet, Sansothy Neth, Bruno Spire, Vohith Khol, Laurent Ferradini, Saramony Sarun, Chandara Mom, Sopheak Ngin, Charlotte Charpentier, Pagnaroat Men, Marion Mora, Vun Mean Chhi, Penhsun Ly, Vonthanak Saphonn

**Affiliations:** ^1^French Agency for Research on AIDS and Viral Hepatitis (ANRS), Paris, France; ^2^University of Health Sciences (UHS), Phnom-Penh, Cambodia; ^3^Institut Pasteur, Paris, France; ^4^France Expertise Internationale, Paris, France; ^5^Aix Marseille Univ, INSERM, IRD, SESSTIM, Sciences Economiques & Sociales de la Santé & Traitement de l’Information Médicale, Marseille, France; ^6^National Center for HIV/AIDS, Dermatology and STD (NCHADS), Phnom Penh, Cambodia; ^7^WHO, Cambodia Office, Phnom Penh, Cambodia; ^8^INSERM, IAME, UMR 1137, Paris, France; ^9^Univ Paris Diderot, Sorbonne Paris Cité, Paris, France; ^10^AP-HP, Hôpital Bichat, Laboratoire de Virologie, Paris, France; ^11^AIDS Healthcare Foundation, Phnom-Penh, Cambodia

**Keywords:** HIV infection, protease inhibitors, adherence counseling, HIV RNA, genotype resistance tests, southeast Asia

## Abstract

**Background:**

Assessment of virological outcomes among HIV-infected patients receiving protease (PR) inhibitor-based second-line regimen are uncommon in Cambodia. The objective of this study is to assess the virological effectiveness of this regimen as well as impact of adherence boosting for patients experiencing virological failure.

**Methods:**

The 2PICAM study (Clinicaltrial: NCT01801618) is a cross-sectional study of HIV-infected adults on PR inhibitor-based second-line regimen since at least 6 months, conducted in 13 representative sites, comprising more than 90% of the target population. Adults with HIV RNA above 250 copies/mL (threshold of the assay) at inclusion received boosted adherence counseling during 3 months followed by HIV RNA control. For confirmed virological failure, genotype resistance test was performed and expert committee used results for therapeutic decision.

**Results:**

Among the 1,317 adults enrolled, the median duration of second-line regimen was 5 years. At inclusion, 1,182 (89.7%) patients achieved virological success (<250 copies/mL) and 135 (10.3%) experienced a virological failure (>250 copies/mL). In multivariable analysis, factors associated with virological success were: CD4 cell count between 201 and 350/mm^3^ (OR: 4.66, 95% CI: 2.57–8.47, *p* < 0.0001) and >350/mm^3^ (OR: 6.67, 95% CI: 4.02–11.06, *p* < 0.0001), duration of PI-based regimen >2 years (OR: 1.64, 95% CI: 1.03–2.62, *p* = 0.037), ATV-containing regimen (0R: 1.65, 95% CI: 1.04–2.63, *p* = 0.034) and high level of adherence (OR: 2.41, 95% CI: 1.07–5.41, *p* = 0.033). After adherence counseling, 63 (46.7%) patients were rescued while 72 (53.3%) were not. For the 54 patients with genotype resistance tests available, high or intermediate levels of resistance to lopinavir, atazanavir, and darunavir were reported for 13 (24%), 12 (22.2%), and 2 (3.7%) patients, respectively. Change to an alternative PR inhibitor-based regimen was recommended for 17 patients and to third-line regimen, including integrase inhibitors for 12.

**Conclusion:**

This study reports high rate of virological suppression of second-line regimen and importance of adherence boosting prior to deciding any change of ART regimen. Genotype resistance tests appear necessary to guide decisions. Such information was of great importance for National HIV Program to adapt guidelines and program needs for third-line regimen.

## Introduction

Global ART coverage among estimated number of individuals living with HIV had reached approximately 41%—or 15 million people—by March 2015 (1). As of end of 2011, 13 countries, including Cambodia, were providing ART to at least 80% of the people estimated to be eligible for HIV treatment. In Cambodia, the number of adults and children on first-line ARV regimen increased dramatically these last 6 years, reaching 54,769 by the end of 2015 (2). Globally, the effectiveness of WHO-recommended first-line regimen was assessed in several studies with good outcomes in adults (3–5) and children (6) related to a high level of adherence (7, 8). In 2011, good outcomes were also reported with lopinavir (LPV/r)-based regimen in a small study of 70 patients after 24 months of follow-up (9). Since then, no assessment at a larger scale and including atazanavir (ATV/r) regimen has been performed in Cambodia.

In 2010, a multi-cohort analysis of 27 ART programs in resource-limited settings [only four with routine viral load (VL) monitoring] including three countries of Southeast Asia (Cambodia, Myanmar, and Laos) reported treatment failure, defined as the first diagnosis of clinical, immunological or virological failure, for 19% of patients receiving PI-based second-line therapy for more than 6 months (10). In Vietnam, the cumulative incidence of PI-based treatment failure (as defined clinically or immunologically) by 1, 2, 3, and 4 years were 13.1, 18.6, 20.4, and 22.8%, respectively (11). More recently, the TREAT Asia HIV Observational Database reports outcomes of 302 patients under PI-based regimen followed for more than 6 months (12) from different Asian sites with a rate of mortality of 1.1 deaths per 100 patients/year and an overall rate of second-line treatment failure of 8.8 failures per 100 patients/year. As the definition of treatment failure relying on CD4 cell count and clinical evaluation underestimates true virological failure rates, little data about virological second-line treatment outcomes are in fact currently available. Such information is of great importance for National HIV Programs to prevent failures of second-line regimen and anticipate the needs of third-line regimen.

The present study reports the results of a nationwide cross-sectional study aimed to assess the virological effectiveness of PI-based second-line regimen in Cambodia as well as the impact of adherence boosting in patients experiencing virological failure. HIV drug resistance mutations in patients experiencing confirmed virological failure despite adherence boosting are also described and options for alternative second-line or third-line regimen are discussed.

## Materials and Methods

### National Guidelines in Cambodia

At the time of the study in 2013, according to WHO recommendations, the first-line ART regimen in Cambodia consisted of two nucleoside reverse-transcriptase inhibitors (NRTIs) plus a non-nucleoside reverse-transcriptase inhibitor (NNRTI) and TDF + 3TC + EFV as a fixed-dose combination was recommended as the preferred option (13). HIV RNA VL was recommended as the preferred monitoring approach to detect and confirm ART failure and was free of charge for patients (13). Samples were transported to the laboratory of NCHADS where the VL was performed. HIV RNA VL was performed 6 and 12 months after ART introduction and yearly. Treatment failure after at least 6 months of using ARV drugs was defined by a detectable VL above 1,000 copies/mL persisting after 3 months of adherence boosting. Second-line ART regimen for adults consisted of two NRTIs plus a ritonavir-boosted protease inhibitor (PI/r). Heat-stable fixed-dose combination of ATV/r was the preferred boosted PI options for second-line ART regimen since 2012. For the second-line NRTI backbones, the use of fixed-dose combination was recommended as the preferred approach, with at least one NRTI never taken by the patient.

### Study Design and Patient Enrolment

The ANRS 12276 2PICAM study (Clinicaltrial ID: NCT01801618) is a cross-sectional assessment conducted in 13 representative ART sites (6 in Phnom Penh and 7 in province), selected because they displayed more than 30 adult patients on PI-based regimen. Overall, second-line regimen patients from all these sites comprised more than 90% of the total number of adult patients on PI-based regimens in Cambodia. At each site, patients were exhaustively enrolled if they were HIV-infected adults (age > 18 years old), on PI-based second-line regimen since at least 6 months and if they accepted to participate to the study.

### Data Collection and Virological Analysis

Patient recruitment began on February 2013 and ended on April 2014. Data about clinical events and previous antiretroviral regimen were collected retrospectively and reported on the Case Reporting Form the day of inclusion. Samples for CD4-cell count and HIV RNA VL testing were collected.

HIV RNA VL was done at NCHADS laboratory by using the G2 Generic HIV-1 VL ANRS kit (Biocentric, Bandol, France). HIV RNA was extracted from plasma using an Arrow extractor (Nordiag). The real-time PCR Detection was conducted on the ABIprism 7000 (Applied Biosystems). By using 0.2 mL of plasma and Qiagen extraction, the threshold of this assay is 250 copies/mL. Bulk sequencing of reverse transcriptase (RT), protease (PR), and integrase (IN) genes were initially performed at the Institute Pasteur of Cambodia and later at NCHADS’s laboratory. Nested-PCR amplified fragments were sent to the Macrogen Company (Macrogen Inc., Seoul, Republic of Korea) for sequencing. Chromatograms, sent back electronically (around 3 days later) were verified and analyzed using Ceq2000 (Beckman Coulter) software. Nucleotides and amino acid sequences were compared with the HXB2 reference sequence using Mega 4 software. Resistance-associated mutations (RAMs) and drug resistance analysis were performed using GREG2010 software according to ANRS algorithms (http://www.hivfrenchresistance.org).

### Management of Patients with Detectable VL

Adults with detectable VL at inclusion (>250 copies/mL) received boosted adherence counseling every month during 3 months according to national guidelines and returned for VL control at month 4 (VLM4). For those patients, the aim of the three interviews with the psychosocial counselor will be to correct and/or strengthen their adherence to treatment. Virological failure was defined as a VLM4 still above 250 copies/mL after adherence boosting. In case of confirmed virological failure, genotype analysis including RAMs and drug resistance analysis were sent to a Clinical and Virological Expert Committee (CVEC) in charge of providing recommendations to clinicians about the most appropriate ART regimen to propose to each patient.

### Adherence Assessment

Adherence to ART was measured using a validated scale comprising 14 items. In this scale, patients provided information about the doses taken during the 4 days preceding the survey, about whether they respected the dose schedule during the previous 4 days and 4 weeks, and whether treatment interruption had occurred for at least two consecutive days within the previous 4 weeks. The algorithm proposed by Carrieri et al. (14) was used to calculate the adherence score corresponding to the 4 days preceding the survey, by comparing the number of pills taken with those prescribed. This allowed us to classify patients into three categories according to adherence level: high adherence (score = 100%), moderate adherence (score = 80–99%), and low adherence (score < 80%). We used one item evaluating how the dose schedule had been respected. Patients reporting treatment interruption for at least two consecutive days were included in the low-adherence group.

### Ethical Issues

Patients were enrolled if they have signed a consent form after receiving appropriate study information. The study was approved by the National Ethics Committee for Health Research in Cambodia and the “Pole Intégré de Recherches Cliniques” (PIRC) of Pasteur Institute (Paris).

### Statistical Analysis

The primary outcome was the prevalence of virological success at inclusion, defined as an HIV VL < 250 copies/mL. The secondary outcomes were 1/the proportion of patients in virological success (<250 copies/mL) after 3 months of boosted-adherence counseling among those in virological failure at inclusion 2/the prevalence of drug resistance defined by the presence of RAMs inducing resistance according to the ANRS algorithm to one or more drugs in the standard second-line regimen(s) used in Cambodia.

Potential explanatory variables included age, sex, CD4 cell-count, duration, and type of PI-based regimen and adherence level.

First, a univariable logistics regression analysis was conducted to examine the relationship between virological success at inclusion and each potential explanatory variable. Then, a multivariable logistic regression model was conducted by including those factors with evidence of an association in the univariable analysis and a *P* value of <0.2. Age and sex were kept in the final model as they were *a priori* specified confounders. Stata software version 14.2 (StataCorp, College Station, TX, USA) was used for all statistical analysis.

## Results

**Figure 1:**
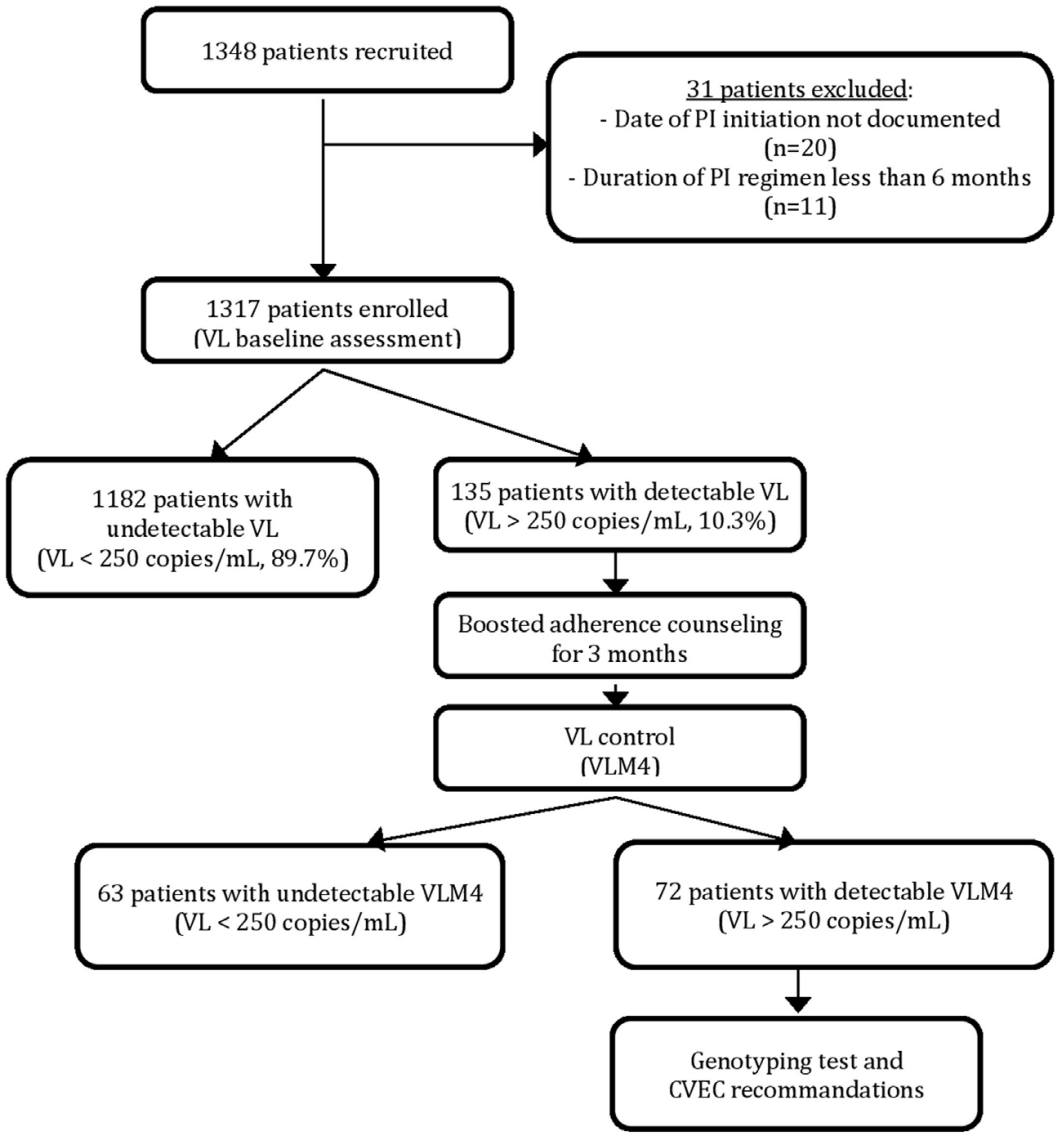
Patient enrolement and virological assessment.

Among the 1,348 PI-based regimen patients recruited for the study, 31 were excluded because of non-eligibility criteria and 1,317 patients were enrolled for evaluation. Results of the VL assessment at inclusion revealed that 1,182 patients (89.7%) had VL below 250 copies/mL and 135 (10.3%) had a VL above 250 copies/mL (Figure 1).

Data at inclusion in the study for eligible patients are reported in Table 1. The median age was 42 years old (IQR, 37–48) and 801 (61%) patients were males. The median duration of PI-based regimen was 4.98 years (IQR, 2.7–6.7). CD4 cell count was above 350/mm^3^ in 75% of patients. Adherence level was high for 83% of patients.

**Table 1:** Characteristics of patients at inclusion in the study (*N* = 1,317).

	Total patients
**Gender**	
Female	516 (39.2%)
Male	801 (61.8%)
**Age at inclusion^a^ (years)**	
Age, median (IQR)	42 (37–48)
**Baseline CD4 cell-count at inclusion (cells per µL)^b^**	
Mediane, IQR	496 (354–677)
<200	99 (7.6%)
201–350	222 (17%)
>350	986 (75.4%)
**Duration of PI-based regimen (years)**	
Median, IQR	4.98 (2.74–6.67)
<2	204 (15.5%)
>2	1,113 (84.5%)
**Second-line regimen^c^**	
LPV-containing	193 (14.8%)
ATV-containing	1,090 (83.5%)
ATV + LPV-containing	23 (1.7%)
**Adherence level^d^**	
Low adherence	41 (3.1%)
Moderate adherence	178 (13.5%)
High adherence	1,096 (83.4%)

*^a^N = 1,304*.

*^b^N = 1,307*.

*^c^N = 1,306*.

*^d^N = 1,315*.

### Characteristics of PI-Based Regimen Patients with VL > 250 copies/mL

The median age of patients with a VL above 250 copies/mL was 42 year-old (IQR: 35–48), and 87 (64.4%) were males (Table 2). VL at inclusion was below 100,000 copies/mL for 105 (77.8%) of them. The median CD4 was 360 cells/mm^3^ (IQR: 165–492), and 29.9% of these patients were in an advanced stage of immunosuppression with CD4 below 200 cells/mm^3^. The duration on PI-based regimen was 3.9 years (IQR: 1.6–6.2) at the time of inclusion, and 97 patients (71.9%) were receiving ATV/r-based regimen while 35 (25.9%) were receiving LPV/r-based regimen and 3 (2.2%) an association ATV/LPV/r.

**Table 2:** Factors associated with PI-based second-line regimen success at time of inclusion (*N* = 1,281).

	Viral load (VL) failure (*N* = 135)	VL success (*N* = 1,182)	Univariate OR (95% CI)	*p* Overall	Multivariate OR (95% CI)*N* = 1,281	*p* Overall
**Gender**						
Female	48 (35.6)	468 (39.6)	1		1	
Male	87 (64.4)	714 (60.4)	0.84 (0.58–1.22)	0.36	0.93 (0.62–1.41)	0.76
**Age (years)^a^**						
Age, Median (IQR)	42 (35–48)	42 (38–48)		0.10		0.34
**Baseline CD4 (cells per µL)^b^**						
Mediane, IQR	360 (165–492)	517 (376–688)		<0.0001		
<200	40 (29.9)	59 (5)	1		1	
201–350	25 (18.7)	197 (16.8)	**5.34 (3–9.52)**	**<0.0001**	4.66 (2.57–8.47)	<0.0001
>350	69 (51.5)	917 (78.2)	9.01 (5.63–14.42)	<0.0001	6.67 (4.02–11.06)	<0.0001
**Duration of PI-based regimen (years)**						
Mediane, IQR	3.88 (1.57–6.19)	5.08 (2.88–6.70)		**0.0001**		
<2	40 (29.6)	164 (13.9)	1		1	
>2	95 (70.4)	1,018 (86.1)	**2.61 (1.74–3.92)**	<0.0001	**1.64 (1.03–2.62)**	**0.037**
**Second-line regimen^c^**						
LPV	35 (25.9)	158 (13.5)	**1**		1	
ATV	97 (71.9)	993 (84.8)	**2.27 (1.49–3.46)**	**0.0014**	**1.63 (1.02–2.59)**	**0.039**
ATV + LPV	3 (2.2)	20 (1.7)	1.48 (0.41–5.24)	0.55	1.26 (0.33–4.77)	0.74
**Adherence level**						
Low adherence	11 (8.1)	30 (2.6)	1		1	
Mod adherence	17 (12.6)	161 (13.6)	3.47 (1.48–8.14)	**0.004**	2.37 (0.92–6.10)	0.073
High adherence	107 (79.3)	990 (83.8)	3.39 (1.65–6.96)	**0.001**	**2.41 (1.07–5.41)**	**0.033**

*^a^N = 1,304*.

*^b^N = 1,307*.

*^c^N = 1,306*.

*Significant p value are in bold font*.

### Factors Associated with PI-Based Second-Line Regimen Success in Univariable and Multivariable Logistic Regression Analysis

Factors associated with PI-based second-line regimen success at time of inclusion are reported in Table 2. In multivariable analysis, factors associated with virological success are: CD4 cell count between 201 and 350/mm^3^ (OR: 4.66, 95% CI: 2.57–8.47, *p* < 0.0001) and >350/mm^3^ (OR: 6.67, 95% CI: 4.02–11.06, *p* < 0.0001) compared to CD4 cell-count <200/mm^3^, duration of PI-based regimen >2 years (OR: 1.64, 95% CI: 1.03–2.62, *p* = 0.037) compared to duration <2 years, ATV-containing regimen (OR: 1.63, 95% CI: 1.02–2.59, *p* = 0.039) compared to LPV-containing regimen and high leve of adherence (OR: 2.41, 95% CI: 1.07–5.41, *p* = 0.033) compared to low level of adherence.

### VL Control after Boosted Adherence Counseling

After 3 months of boosted adherence counseling, a VL control (VLM4) was performed for all patients with detectable baseline VL (Figure 1). Among them, 63 (46.7%) patients had a VLM4 undetectable while for 72 (53.3%) VLM4 remained detectable. These later patients were considered as confirmed PI-based regimen virological failures and represented 5.⋅47% of the total enrolled patients. Their median VLM4 was 3,417 copies/mL (IQR: 870–28,403), and nine (12.⋅5%) patients had VLM4 > 100,000 copies/mL.

### HIV-1 Genotyping Analysis of PI-Based Second-Line Regimen Patients with Confirmed Virological Failure

**Figure 2:**
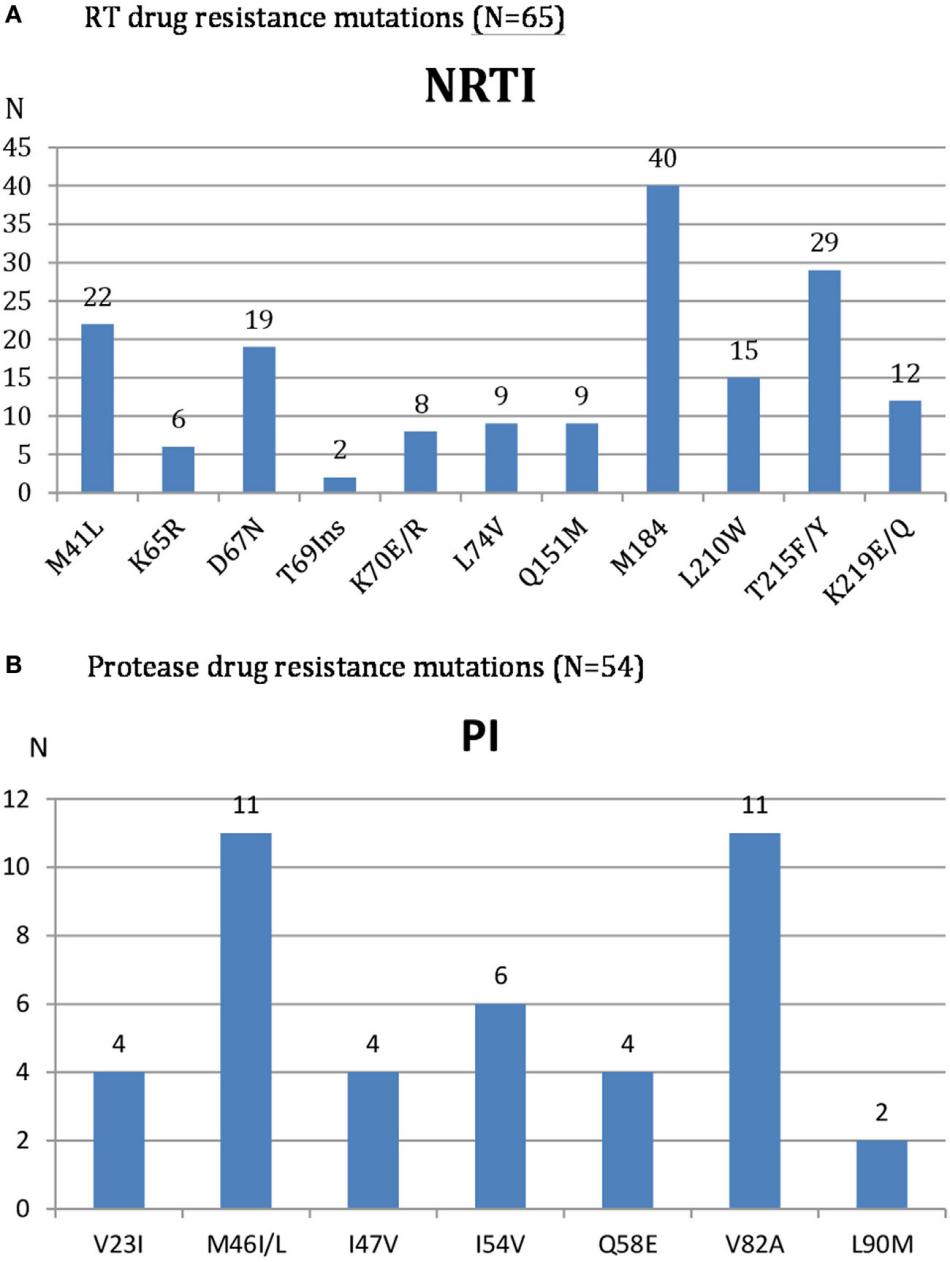
HIV Reverse transcriptase **(A)** and protease **(B)** drug resistance mutations among patients with confirmed virological second time treatment failure.

Genotypic resistance test (GRT) was performed in all 72 patients with confirmed virological failure after 3 months of adherence boosting. The description of observed RT and PR drug resistance mutations is reported in Figure 2.

Reverse transcriptase sequences were available in 65 samples because of 7 PCR amplification failures. According to ANRS resistance algorithm, high or intermediate level of resistance to AZT and TDF was reported in 35 (53.8%) and 20 (30.8%) of them, respectively.

Protease sequences were available in 54 samples because of 18 PCR amplification failures. High or intermediate level of resistance to LPV/r, ATV/r, and DRV/r was reported in 13 (24.0%), 12 (22.2%), and 2 (3.7%) patients, respectively. Globally, 20 (37%) patients had resistance to PI (all were also resistant to NRTI), and 34 (63%) did not present resistance to PI (13 of them displayed also resistance to NRTI).

### Advised Alternative Regimen for Patients with Confirmed Second-Line Virological Failure

For the 54 patients with GRT available including PR mutations, the recommendation of the CVEC was to continue current regimen for 25 (46.3%) patients (absence of resistance), change to an alternative PI-based regimen using drugs available in Cambodia (ATV/r, LPV/r, TDF, AZT, ddI, ABC) for 17 (31.5%) and change to a needed third-line regimen including IN inhibitors for 12 (22.2%) patients.

For the six patients without GRT available for either RT or PR, no change of ART regimen was recommended.

For the 12 patients with available GRT for RT but not for PR, the recommendation of the CVEC were to not change for seven patients (absence of resistance), change with available drugs for three patients, and change with an association including a PI/IN inhibitors for two patients because of resistance to all NRTI.

## Discussions

The present study reports a high rate of virological suppression for HIV-infected adults on PI-based second-line regimen in Cambodia with 90% of patients in virological success at time of inclusion. In addition, almost half of those with a detectable VL could be rescued following 3-months of boosted adherence counseling giving a final 94.5% success rate under PI-based regimen. Higher success rates at baseline were observed in patients with high CD4 cell count, longer duration of PI-based regimen, ATV-containing regimen and high level of adherence. Overall, 5.5% of patients on PI-based regimen had confirmed virological failure in Cambodia and 12 of them were already in urgent need of third-line regimen. Such information was of great importance for the National HIV Program to adapt HIV management guidelines and program the needs for alternative second- and third-line regimen.

Similar positive virological outcomes have been previously reported in Cambodia on a small cohort of 70 patients under lopinavir-based regimen (9) and in Thailand on a cohort of 95 patients (15). The present study, however, was designed to be representative of all adults comprising almost 90% of patients on PI-based regimen in the country and confirms these positive outcomes. A meta-analysis of studies conducted in resource-limited settings reported a pooled prevalence of virological failure of 23.1% after 12 months of treatment with PR-inhibitor-based regimen (16). More recently, a cohort study in South Africa reported a prevalence of virological failure of 17.8% after 6 months of treatment (17). In our study, the prevalence of virological failure among the 204 patients with treatment duration below 2 years is 20%, which is consistent with the previous data and highlight the fact to improve the early detection of these patients.

The control of adherence is a critical factor of ART success and was significantly associated with virological success in our study. WHO recommends adherence support intervention prior to deciding any change of ART regimen. Indeed, it was found that a significant number of patients could be rescued after initial identification of treatment failure but the effectiveness of such a strategy still remains unclear. We report such adherence boosting strategy is effective among PI-based second-line patients with 46.7% patients with detectable VL being rescued having an undetectable control VL after 3 months. Following this result, HIV national program has revised in 2017 the national guidance to enhance ART adherence. Similar results of adherence boosting strategy were reported in India where 34% of PI-based regimen patients with virological failure had a suppressed VL after 6 months (18) and more recently in South Africa (17, 19). However in our study, among the 72 patients who could not be rescued and had confirmed VL failure, 21 patients presented no resistance to PI or NRTI drugs and should have been rescued if adherence counseling was fully effective. This shows that adherence-counseling intervention still have room for improvement to be fully effective. For this purpose, a better understanding of individual and structural factors associated with low adherence in the Cambodian context is critical and were recently analyzed (20).

When analyzing factor associated with virological success at time of inclusion, we found that having a high baseline CD4 cell count (above 200/mm^3^), being on ATV/r containing regimen, having a high adherence level and a longer duration of PI-based regimen were independently associated with success. The longer delay of PI-based regimen associated with success might be surprising but could suggest that higher risk of failures and death occurred early after PI-based regimen initiation, as it was reported in India (18). The majority of patients were switched from lopinavir to ATV/r-containing regimen before the implementation of the study, as ATV/r is distributed as a generic drug combining atazanavir and ritonavir in one pill once a day and as such changing strategy was reported to be effective elsewhere. The fact that ATV/r-containing regimen was independently associated with success in our study might reflects the benefits in term of adherence of such once-daily regimen compared to more than one daily intake as already described (21). In addition, fewer gastrointestinal adverse events were also reported for ATV/r compared to LPV/r (22) also contributing to improve adherence (23).

Analysis of HIV-1 mutations of patients with confirmed virological failure revealed that even after almost 4 years median duration on PI-based regimen, the accumulation of RT and PR drug resistance mutations was relatively low. The main observed RAMs were M184I/V, T215F/Y, M41L, and D67N for RT and M46I/L and V82A for PR. These PR mutations are similar to those reported in Nigeria (24) and Vietnam (21) which confer high level of resistance to LPV with risk of accumulations overtime (24) and worsening outcomes (25). The moderate prevalence of PR inhibitor resistance reported in Sub-Saharan Africa (26) was confirmed in Cambodia as only 37% of patients among those with confirmed virological failure had resistance to PI while the remaining (63%) did not. For some patients, it was possible to continue the PI used or to switch for LPV to ATV and conversely while for the others, DRV was the only PI option. In two cases, resistance to DRV was observed and no PI option was available. The national program is now actively working to have DRV available in Cambodia as an alternative second-line regimen.

Given the still poor affordability of third-line ARV drugs, GRT seems to be necessary and important before deciding to switch to third-line regimen. WHO recommends switching to third-line regimen (DRV/r + IN inhibitor with or without 1-2 NRTI) for patients failing second-line regimen if VL remains above 1,000 copies/mL after 3 months of adherence boosting. In our study, among the 72 patients with confirmed VL failure after adherence boosting, 52 had a VL above 1,000 copies/mL and should have been switched to a third-line regimen. However, according to the results of GRT, only 12 were really in need of third-line regimen, which should include an IN inhibitor. The majority of the remaining patients can continue their current regimen or could benefit from adapted alternative second-line regimen. Because of the high cost of third-line drugs, the use of HIV GRT before switching to third-line regimen could be a cost-effective intervention, which needs further investigation. WHO recently recommend expansion of HIV drug resistance test and encourage innovative research in this topic to have the greatest public health impact (27). The use of point-of care GRT focusing on few major PI-RAMs (as M46I, I54V, and V82A) could be useful to reduce cost and improve accessibility, as described for NRTI and NNRTI RAMs (28).

Integrase inhibitors are essential components of third-line regimen but both raltegravir (RAL) and dolutegravir (DTG) are not currently available in Cambodia. The choice of one of these drugs will be important for National program awaiting further recommendations. RAL have shown favorable outcomes in association with LPV/r (29, 30) but it needs to be taken twice daily which could impact adherence and resistances could appear quickly in case of VL failure (31). On the other hand, DTG, also recommended by WHO as an alternative for first-line regimen, represents an interesting option as it could be taken once daily, displays high genetic barrier to resistances, and a first generic version was recently approved. However, few data are still available for its use in association with PI in resource-limited setting. Recently, a retrospective study had reported a high proportion of viral suppression even in highly experienced HIV-1-infected patients (32). More studies are necessary to assess the effectiveness of the DTG/PI association in resource-limited setting. Following the results of the 2PICAM study, a non-comparative multicenter pilot prospective study was just accepted for funding by ANRS in July 2017 with the objective to assess the virological effectiveness of a third-line regimen combining DTG, ritonavir-boosted darunavir (DRV/r) and optimized NRTI recycling.

Our study had some limitations. First, this is a cross-sectional study and we are not able to provide data on long-term follow-up, especially for patients with confirmed virological failure. Second, this study was limited to adults HIV patients and data about the effectiveness of second-line regimen in children and adolescents are still pending. Third, the findings related to the type of PI used could be due to bias by indication.

In conclusion, this study reports the high rate of virological suppression of PI-based second-line regimen in Cambodia nation wide and the importance of boosted adherence counseling prior to deciding any change of ART regimen. Such a very high virological success rate of PI-based regimen after a median duration of 5 years reveals the strength of such regimen and is very encouraging concerning the duration of available PI-based second-line regimen.

HIV genotyping appears necessary to guide decisions for alternative second-line or third-line regimen. IN inhibitors are key components of potential third-line regimen, and DTG could represent an interesting option. Results of this study were of great importance to adjust HIV national guidelines and to pave the way to a further assessment of a third-line regimen option in Cambodia.

## Ethics Statement

Patients were enrolled if they have signed a consent form after receiving appropriate study information. The study was approved by the National Ethics Committee for Health Research in Cambodia (NECHR) and the “Pole Intégré de Recherches Cliniques” (PIRC) of Pasteur Institute (Paris).

## Author Contributions

Design of the study: EN, SAN, BS, VK, LF, MCV, PL, and VS. Collection of data: EN, SAN, VK, BS, and SS. Virological analysis: EN, CM, SON, and CC. Data interpretation: all authors. Data analysis: OS, EN, LF, BS, and MM. Writing committee: OS, EN, BS, LF, CC, and VS. Revising the work: all authors. Final approval of the version: all authors. Agreement to be accountable for all aspects of the work: all authors.

## Conflict of Interest Statement

The authors declare that the research was conducted in the absence of any commercial or financial relationships that could be construed as a potential conflict of interest.
